# The Sufferings of Christ

**Published:** 1888-05-12

**Authors:** 


### WORDS OF CONSOLATION.

[Communications for this column should be addressed "Chaplain," care of the Editor of THE HOSPITAL, who will gratefully welcome any suggestions or inquiries from matrons, nurses, and the staff generally.]

### THE SUFFERINGS OF CHRIST.—LXXVI.

*For Reading to the Sick.*

WITH this paragraph we shall bring our readings upon this subject to a close. It is only necessary to observe that the continued outpouring of our Blessed Lord's life for the preservation of our life eternal is the fruit of His unselfishness. It is, indeed, the continuation of His unselfishness, the continuation of sacrifice, not as propitiation, but in the sense of still giving forth virtue from His person. Shall we limit His self-sacrifice to suffering? Oh no. He is ever giving Himself for us, and to us if we will receive Him. But this involves no pain: it is a great pleasure to Him now. Why? Because He died unto self so completely once. Therefore the seed hath risen in all its beauty and beareth much fruit. We can follow Him here, though it may be at a distance, and be thankful. The law is ever the same. The only lives which are really useful and permanently fruitful are those which have first fallen to the earth and died to themselves. These, through mortifying all carnal and selfish affections, arise to a higher and nobler life; yea they arise with spiritual natures even here, with something about them of their Master's power of diffusion for the sake of others. From dying unto self, in addition to dying unto sin, they arise to give themselves for others and to others; and so they bring forth much fruit, which their brethren and sisters partake of and live by in their turn. Only in so far as we die unto self are we really serviceable to others. Selfishness means being wrapped up in self, and thus wrapped up to abide alone. The dissolution of the old nature has not taken place in the selfish. The husk is still clinging. How can such an existence benefit others? "But if it die it bringeth forth much fruit." Wherever you see self-sacrifice, there the essence of the Cross is spreading. Said St. Paul, "I die daily." (1 Cor. xv. 31.)

